# A Systematic Review of Dental Antibiotic Stewardship Interventions

**DOI:** 10.1111/cdoe.13009

**Published:** 2024-10-14

**Authors:** Leanne Teoh, Christin Löffler, Michelle Mun, Anirudha Agnihotry, Harpinder Kaur, Karen Born, Wendy Thompson

**Affiliations:** ^1^ University of Melbourne Carlton Victoria Australia; ^2^ Universitätsmedizin Rostock Rostock Germany; ^3^ Stevenson Dental Research Institute San Dimas California USA; ^4^ Western University London Ontario Canada; ^5^ University of Toronto Toronto Ontario Canada; ^6^ University of Manchester Manchester UK

**Keywords:** antibiotics, antimicrobial resistance, antimicrobial stewardship, dental, dental prescribing, primary care

## Abstract

**Background:**

Antimicrobial resistance is a significant threat to global health. Antimicrobial stewardship is reducing inappropriate antimicrobial prescribing to counter it. Dentists prescribe ~10% of all antibiotics worldwide, yet up to 90% of antibiotic prescriptions by dentists are inappropriate. The aim of this systematic review was to update a 2017 review evaluating the effects of antibiotic stewardship interventions in dental settings, using the international consensus on core outcomes for dental antibiotic stewardship.

**Methods:**

Systematic database searches were undertaken in April 2023, of the: Cochrane Oral Health Group Trials Register, Cochrane Central Register of Controlled Trials, MEDLINE via OVID, EMBASE via OVID, Dentistry and Oral Sciences Source, the US National Institutes of Health Trials Register, the World Health Organisation International Clinical Trials Registry Platform and the ISRCTN registry databases. Randomised controlled trials (or non‐randomised studies with clearly reported mechanism of group formation and inclusion criteria) of interventions to optimise and/or reduce dental antibiotic prescribing were eligible for inclusion. Two authors independently screened for eligible studies. Risk of bias was assessed using the Cochrane Risk of Bias 2 tool, certainty of evidence assessed using GRADE. Meta‐analysis was planned whether the results of studies reported similar outcomes, otherwise narrative synthesis was undertaken.

**Results:**

Three eligible studies randomising 2148 participants were included. The interventions were combinations of education, audit and feedback and written behaviour change messages, guideline summary, practice visits and patient leaflets. None of the control groups received an intervention. All three included studies measured the quantity of antibiotics prescribed and two measured the appropriateness of prescribing. None measured patient‐reported or adverse outcomes. Two included studies were assessed as ‘high risk’ and one with ‘low risk’ of bias. There was high‐certainty evidence that audit and personalised feedback with individualised behaviour change messages can be effective. Evidence for in‐person education was low‐certainty. Guideline dissemination alone was ineffective at improving antibiotic prescribing. Due to different outcomes reported, meta‐analysis was inappropriate.

**Conclusion:**

Although various dental antibiotic stewardship interventions have been reported in the literature, only three have been evaluated using a randomised design, of which only one provided high certainty evidence. To strengthen the body of evidence, well‐powered, robust, randomised controlled trials are required, with adequate follow‐up, reporting the internationally‐agreed core outcomes and including a parallel process evaluation is recommended.

**Trial Registration:** PROSPERO (CRD42023411476)

## 1 Introduction

Antimicrobial resistance is an increasing global public health problem [1]. In 2019, ~2 million deaths were attributed to infections resistant to antibiotics, more than HIV and malaria—making antibiotic resistance (AMR) one of the biggest killers [1]. Left unchecked, each year AMR could kill 10 million people and reduce global GDP by 3.8% by 2050 [2]. Providing appropriate, quality‐assured treatment to optimise antibiotic use across healthcare is critical to addressing the challenge of antibiotic resistance (ABR) [3]. Dental antibiotic prescribing accounts for a significant 10% of all prescriptions across human healthcare worldwide, including both therapeutic and prophylactic indications [4].

Significant evidence of inappropriate antibiotic prescribing by dentists in high‐, middle‐ and low‐income countries exists, with up to 90% in UK, Lebanon and Ghana prescribed not in accordance with guidance [5, 6, 7]. Antibiotic stewardship (ABS) interventions to optimise the use of antibiotics in dentistry are thus urgently needed [8] and have been highlighted by several global and international organisations. The World Health Organisation's recently published Global Oral Health Status Report, the FDI World Dental Federation's White Paper regarding the essential role of dental teams in tackling ABR, and the Australian Commission on Safety and Quality in Health Care all highlight this important need [8, 9, 10].

A systematic review of dental ABS interventions, published in 2017, identified nine studies for inclusion, of which most were assessed as low quality due to the design (such as no randomisation or control group) or lack of information about study methodology [11]. It highlighted the importance of higher‐quality research with objective and standardised outcome reporting, longer periods of follow‐up, rigorous methodology and adequate standard of study reporting [11]. Since 2017, a number of studies have been published which demonstrate interventions tailored for dentistry can improve and/or reduce dental antibiotic prescribing [12, 13, 14]. An international consensus on a core outcome set for dental ABS has also been published [15]. The core outcome set focuses on optimised antibiotic prescribing by measuring both quantity and quality of antibiotic prescribing, as well as rates of adverse outcomes and a patient‐reported outcome about normalcy of function.

This systematic review aims to update the 2017 review by overcoming its limitations and including only research with more rigorous methods, such as studies with randomisation and control groups, and using the international consensus on a core outcome set for dental ABS, to evaluate the effects of interventions to optimise the prescribing of antibiotics in dentistry.

## 2 Methods

This systematic review was registered in PROSPERO (CRD42023411476) and the Cochrane Handbook for Systematic Reviews of Interventions v6.3 was followed [16]. The study was reported in accordance with the PRISMA 2020 Statement [17].

### 2.1 Eligibility Criteria

#### 2.1.1 Participants

Adults and children receiving general or specialist care services in dental settings were eligible for inclusion. Non‐dental settings were excluded.

#### 2.1.2 Interventions

Interventions designed to optimise or reduce dental antibiotic prescribing which targeted clinicians or patients, were included. Studies without interventions were excluded.

#### 2.1.3 Comparators

Studies with a clearly defined comparator group, including those comparing an active intervention to standard care, usual practice, or no intervention were eligible for inclusion, as were studies comparing one active intervention with another. Studies without a comparator were excluded.

#### 2.1.4 Outcome measures

Outcomes included were quantity (rate) and quality (appropriateness) of antibiotics prescribed, adverse outcomes and the patient‐reported normalcy of function, defined as an individuals' ability to carry on with daily life as normal. Treatment efficacy, knowledge, attitudes and/or intention to prescribe were excluded.

#### 2.1.5 Studies

Randomised controlled trials and non‐randomised studies with a clearly defined comparator group were eligible for inclusion. Other study types were excluded.

There were no restrictions based on language or date of publication.

### 2.2 Information Sources

The following databases were searched on 3 April 2023 from their earliest dates: Cochrane Oral Health Group Trials Register (whole database), Cochrane Central Register of Controlled Trials (CENTRAL) (The Cochrane Library, current issue), MEDLINE via OVID (1946 to present), EMBASE via OVID (1980 to present), Dentistry and Oral Sciences Source. The US National Institutes of Health Trials Register (http://ClinicalTrials.gov), the World Health Organisation (WHO) International Clinical Trials Registry Platform (http://apps.who.int/trialsearch/default.aspx) and the ISRCTN registry (https://www.isrctn.com/) databases for ongoing trials were also searched. Grey literature was not searched.

#### 2.2.1 Search Strategy

The search strategy was developed with a specialist in systematic reviews from the University of Manchester and is shown in Data S1. No language restrictions were used.

#### 2.2.2 Selection Process

The identified articles were entered into Covidence (www.covidence.org) where duplicates were removed. Two researchers (C.L. and H.K.) independently screened the titles and abstracts for inclusion. Two researchers (L.T. and M.M.) undertook full text screening of the potential studies for inclusion, and discrepancies were resolved through discussion with a third researcher (W.T.). All full text studies that did not meet the inclusion criteria are recorded in Table S2.

#### 2.2.3 Data Collection Process

A data collection form for study characteristics was piloted using the categories: Intervention, intervention components, population, setting, outcomes, methods and funding.

Two researchers (L.T. and M.M.) independently collected data for each included study which is shown in Table 2. The interventions were described in accordance with the behaviour change technique taxonomy version 1 (BCTT v1) [18, 19]. The outcomes were reported according to the international consensus on a core outcome set for dental ABS [15].

#### 2.2.4 Data Items

In accordance with the international consensus on a core outcome set for dental ABS [15], the primary outcomes were measures of antibiotic prescribing by quantity (amount and rate of antibiotic prescribing) and/or quality (appropriateness in accordance with a defined clinical standard). The secondary outcomes were adverse or poor outcomes (serious adverse outcomes, need for escalation of care and harm resulting from disease progression or from antibiotic treatment) and a patient‐reported outcome measure about normalcy of function: an individual's ability to carry on with daily life.

#### 2.2.5 Study Risk of Bias Assessment

The risk of bias assessment for each included study was performed independently by two researchers (L.T. and M.M.), using the Cochrane Risk of Bias tool for cluster‐randomised trials [20] (see Data S3). Discrepancies were resolved through discussion among the researchers.

#### 2.2.6 Effect Measures and Synthesis Methods

Heterogeneity was examined by considering the methodological characteristics of the studies, the participants, the interventions, and the outcomes to assess whether meta‐analysis was appropriate. The results from studies not suitable for inclusion in a meta‐analysis were reported narratively with regard to the PRISMA guidelines with grouping by populations, interventions and outcomes, where possible [16]. Narrative synthesis included all studies not suitable for meta‐analysis, with an appropriate standardised metric, synthesis method and data presentation method selected following exploration of the included studies and in line with Cochrane guidance [21].

#### 2.2.7 Certainty Assessment

A Summary of Findings table was prepared according to the GRADE (Grading of Recommendations Assessment, Development and Evaluation) approach for assessing certainty (or quality) of the evidence. Authors were contacted to obtain any missing data.

## 3 Results

### 3.1 Study Selection

**FIGURE 1:**
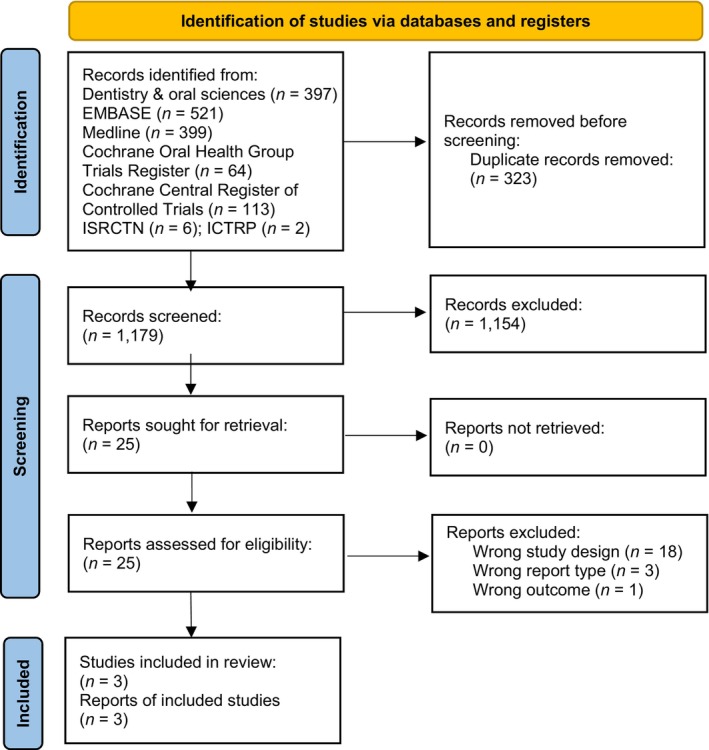
PRISMA flow diagram showing study selection. From Page et al. [17].

Of the 1180 studies identified for potential inclusion, three studies met the inclusion criteria and were included in the final synthesis [22, 23, 24]. The details of the selection process are shown in Figure 1 and the characteristics of the 21 studies excluded following full text assessment are presented in Table S1.

### 3.2 Study Characteristics

Characteristics of the three included studies are detailed in Table 1 [22, 23, 24]. These studies were published between 2006 and 2022 and included general dentists and oral surgeons. The two earlier studies took place in the United Kingdom [22, 23] and the more recent study was in Lebanon [24]. One study reported only on therapeutic dental antibiotic prescribing [22], whilst the other two studies reported on both therapeutic and prophylactic prescribing [23, 24]. Two of these studies were also included in the previous 2017 systematic review [22, 23].

**TABLE 1:** Characteristics of included studies.

Study	Intervention	Population/participants	Setting/context	Outcomes reported	Results	Methods/risk of bias^a^	Funding sources/declaration of interests
Seager et al. (2006) [22]	*Two intervention groups: The first intervention group received guidelines and patient information leaflets by post only; the second received the full intervention: guidelines, patient information leaflets and a visit by the academic detailer* Guidelines: Written guidelines for the management of patients with acute dental pain and a laminated page summary were posted to the participants Patient information leaflets: To which explained that antibiotics are not usually required for such a condition were posted to the participating dentists *Visit by the academic detailer*: To discuss the content of the guidelines and encourage the rational use of antibiotics	Patients: Only those attending with acute dental pain (*n* = 1497) Dentists: One general dental practitioner per study centre. (*n* = 97 recruited and *n* = 70 participated)	NHS general dental practices Four study centres in Wales, UK Study period: Unclear	Primary outcomes: % patients receiving antibiotics % patients receiving antibiotics inappropriately (i.e., absence of facial swelling, lymphadenopathy, trismus, raised temperature, difficulty swallowing, or acute necrotising ulcerative gingivitis) Secondary outcomes: None reported	% antibiotics: Guideline and patient leaflets only: I: 29%; OR: 0.83 (0.55, 1.21) C: 32%; OR: 1 Full intervention: I: 23%; OR: 0.63 (0.41, 0.95) C: 32%; OR: 1 % inappropriate antibiotics (by indication) Guideline only: I: 15%; OR: 0.82 (0.53, 1.29) C: 18%; OR: 1 Full intervention: I: 7%; OR: 0.33 (0.21, 0.54) C: 18%; OR: 1	Randomised controlled trial Comparator: treatment as normal Risk of bias: high	Funded by NHS National Research & Development Programme on Primary Dental Care No declarations of interest reported
Elouafkaoui et al. (2016) [23]	Audit and feedback: A line graph plotting the monthly antibiotic prescribing rate of local dentists and/or the individual dentist was posted to each participant, with or without behaviour change messaging at two different time points: 0 & 6 months, and 0,6 & 9 months	Patients: All seen during the study period (number not specified). Dentists: All providing patient care at the study centres (*n* = 2018 participated)	NHS general dental practices 786 study centres in Scotland, UK Study period: May 2013–April 2014	Primary outcomes: Number of antibiotics per 100 claims Secondary outcomes: None reported	Reduction in antibiotic items per 100 claims All A&F variations I: 1.0 C: 0.4	Randomised cluster controlled trial Comparator: treatment as normal Risk of bias: some concerns	Funded by NHS Education for Scotland No declarations of interest reported
Chehabeddine et al. (2022) [24]	Intervention group consisted of an educational component and a one‐on‐one discussion with a trainer. Educational intervention: oral presentation about evidence‐based recommendations on the use of dental antibiotics, knowledge on bacterial resistance and the misuse of antibiotics, studies showing the appropriate and inappropriate use of antibiotics in dental practice One‐to‐one discussion with a trainer: To discuss the recommendations and their prescribing habits to see how their antibiotic prescribing modalities will change	Patients: All seen during the study period (*n* = 2063) Dentists: General dentists or oral surgeons. 60 recruited and 55 participated	Private dental clinics. Study centres in Beirut and Mount Lebanon (Baabda and Maten) governorates Lebanon: number unclear Study period: April–August 2017	Primary outcomes: % patients receiving antibiotics % prescriptions compliant with guidelines (indication, choice, dose and duration). Secondary outcomes: None reported	**%** antibiotics: I: 11.6% C: 26.0% % guideline compliant (by indication): I: 38.8% C: 15.6%	Randomised controlled trial Comparator: treatment as normal. Risk of bias: high	No funding source reported No declarations of interest reported

^a^ Risk of bias scores detailed in Table 3.

### 3.3 Description of the Interventions

The interventions were combinations of education, practice visits, provision of guideline summaries, patient leaflets and, audit and feedback with personalised graphical and written behaviour change messages, and comparator prescribing information (see Table 2). Each study included a control group which received no intervention. Detailed descriptions of the interventions studied based on the BCTT v1 are presented in Table 2.

**TABLE 2:** Effect direction plot: Summary of intervention impacts on antibiotic prescribing, including vote counting by quantity and quality of prescribing.

Author (year)	Study design	Risk of bias	Description of intervention components (using the behaviour change technique taxonomy v1)	Final sample (int/cont)	Quantity^a^	Quality^a^
Educational intervention—in‐person components						
Seager et al. (2006) [22]	RCT	High	1.4 Action planning 3.1 Social support unspecified 4.1 Instruction on how to perform the behaviour 5.1 Information about health consequences 5.3 Information about social and emotional consequences 9.1 Credible source 12.2 Restructuring the social environment 12.5 Adding objects to the environment 15.1 Verbal persuasion about capability	556/490	**↓**	**↑**
Chehabeddine et al. (2022) [24]	RCT	High	1.4 Action planning 3.1 Social support unspecified 4.1 Instruction on how to perform the behaviour 5.1 Information about health consequences 5.3 Information about social and emotional consequences 9.1 Credible source 15.1 Verbal persuasion about capability	971/1092	**↓**	**↑** ^c^
Educational intervention—remote guideline component only						
Seager et al. (2006) [22]	High	High	4.1 Instruction on how to perform the behaviour 9.1 Credible source	451/490	**→**	Not reported
Audit & feedback—all variations^b^						
Elouafkaoui et al. (2016) [23]	RCT	Some concerns	Audit and feedback (A&F)—all variations 2.2 Feedback on behaviour 9.1 Credible source	1550/438	**↓**	Not reported
Elouafkaoui et al. (2016) [23]	RCT	Some concerns	A&F with BCM vs. no BCM 4.1 Instruction on how to perform the behaviour 5.1 Information about health consequences	766/784	**↓**	Not reported
Elouafkaoui et al. (2016) [23]	RCT	Some concerns	A&F with comparator vs. no comparator No components in addition to the core A&F	793/757	→	Not reported
Elouafkaoui et al. (2016) [23]	RCT	Some concerns	A&F provided at 0, 6 months vs. 0, 6, 9 months No components in addition to the core A&F	740/810	→	Not reported
				VOTE COUNTING	3 ↓ 1 →	2 **↑**

^a^ Quantity = rate of total antibiotic; Quality = reduction in inappropriate antibiotic prescribing.

^b^ All variations of Elouafkaoui et al.'s audit and feedback (A&F) intervention include: (a) A&F graphical intervention (incorporating results from variations b–d); (b) A&F with vs. without behaviour change messaging (BCM); (c) A&F with vs. without comparator information from the local health board; (d) A&F feedback information provided at 0 and 6 months vs. 0, 6, and 9 months. Only the results combining all variations (a) are included within vote counting to prevent double counting.

^c^ Overall figure calculated from detail within the paper (see Table 2).

### 3.4 Risk of Bias Assessment

Two included studies were assessed as having a high risk [22, 24] and one low risk of bias [23]. Concerns with the high risk of bias studies related to selection of the reported results, and missing data related to the collection of individual patient data [22, 24], as opposed to the use of routinely collected data in the low risk study [23]. In addition, the outcomes of one study [22] may have been affected by possible loss to follow‐up. The risk of bias assessment is shown in Data S3. The risk of bias table is shown in Table 3.

**TABLE 3:** Risk of bias assessment (ROB2).

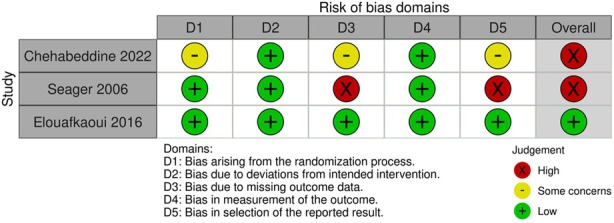

### 3.5 Results of Individual Studies

All studies reported one primary outcome: quantity of antibiotic prescribing. Whilst a variety of quantitative outcome measures were employed, none used exactly the same ones (see Table 2). Two of the studies also included another primary outcome: quality of antibiotic prescribing, measured as appropriateness of the antibiotic prescription in relation to a defined clinical standard [22, 24].

None of the included studies reported any of the study's secondary outcomes. Whilst Seager et al. [22] set out to collect patient‐reported outcomes, it was abandoned due to a slow rate of questionnaire return.

### 3.6 Heterogeneity of the Included Studies

The three included studies encompass a heterogenous variety of intervention components (see Tables 1 and 2), populations (all patients versus just those with acute pain) and reported outcomes (see Table 1). The interventions and reported outcomes were so different that they could not be combined, making meta‐analysis inappropriate.

### 3.7 Results of Syntheses

Narrative synthesis of the results from the three included studies was based on grouping by intervention (educational, and audit and feedback) and vote counting using the standardised metric ‘direction of effect towards the study outcomes’ [21]. An effect direction plot [25] of the results is presented in Table 2. Overall, the direction of effect was towards improved antibiotic prescribing, with reduced quantity demonstrated in all three studies and increased quality in two studies [22, 24].

#### 3.7.1 Interventions Including an Educational Element

Two studies evaluated interventions which included an in‐person educational element, with both demonstrating a direction of effect towards reduced quantity and improved quality of antibiotic prescribing by indication (see Table 2) [22, 24]. Seager et al. [22] demonstrated a reduction in quantity of antibiotic prescribing from 32% in the comparator (usual practice) group to 23% (OR: 0.63 (0.41, 0.95)) in the intervention group, and a reduction in inappropriate antibiotics by indication from 18% to 7% (OR: 0.33 (0.21, 0.54)) in these groups respectively. Chehabeddine et al. [24] similarly demonstrated a reduction in antibiotics by quantity from 26% to 11.6%, and an improvement in guideline compliance by indication from 38.8% in the comparator (usual practice) group and 15.6% in the intervention group. No confidence intervals were reported in this study and authors were contacted for this information without response. Seager and colleagues also evaluated the impact of solely posting guideline information to dentists and showed no difference in the quantity or quality of antibiotic prescribing [22].

#### 3.7.2 Audit and Feedback

One study evaluated an audit and feedback intervention with various elements [23]. Graphical presentation of individual dentist's data together with behaviour change messaging delivered a direction of effect towards reduced antibiotic prescribing (see Table 2). The rate of antibiotic prescribing in the control (usual practice) group reduced by 0.4 antibiotic items per 100 treatment claims. In the intervention group, it reduced by 1.0, representing a 5.7% difference, which was calculated to be statistically significant [23]. Neither the addition of comparator information from the local health board nor inclusion of an additional timepoint for providing information (three times rather than just twice) to the dentists improved the effect.

### 3.8 Certainty of Evidence

There was high certainty that the use of audit and feedback reduced dental antibiotic prescribing [23]. The study by Seager et al. [22] demonstrated low and very low certainty evidence that the intervention resulted in a reduction in antibiotic prescribing and improvement of appropriateness, respectively. There was insufficient information to assess the evidence from Chehabeddine and colleagues [24]. Authors were contacted without response. The SoF table is presented in Table S4.

## 4 Discussion

Three randomised controlled studies were identified which assessed the impact of dental ABS complex interventions on the quantity and quality of antibiotic prescribing [22, 23, 24]. Where the direction of effect could be determined, the quantity of antibiotic prescribing was lower and the quality of prescribing better in the intervention group than in the control group, although the quality of evidence was low to high. All of the studies employed interventions with a variety of components which have been grouped as educational interventions, and audit and feedback interventions [22, 23, 24]. The interventions involving an in‐person educational element and the audit and feedback intervention with addition of a behaviour change messaging component demonstrated a direction of effect towards improved antibiotic prescribing by dentists. Posting guidelines to dentists made no impact on reducing antibiotic prescribing. Similarly, among the audit and feedback variations, providing comparator information and providing feedback on three rather than just two occasions made no additional impact on reduced antibiotic prescribing beyond that from the standard audit and feedback intervention.

The Centres for Disease Control and Prevention (CDC) recommends a range of core elements for outpatient ABS, including education and expertise and ‘tracking and reporting’ (also known as audit and feedback) [26]. The three studies included in our systematic review align with these two CDC elements. The CDC suggests two additional elements ‘commitment’ and ‘action for policy and practice’. Many of the studies excluded from our review due to study design without a comparator reported studies which align with these core CDC elements. Gross et al. [14] addressed ‘commitment’, and online prescriber decision support [12] and the use of biomarker testing [27] to assist diagnosis are examples of ‘action for policy and practice’. Translation of ABS interventions from other healthcare contexts to dentistry have been proposed [28], although careful consideration of the context has been highlighted as a systematic review of factors influencing antibiotic prescribing for acute conditions across primary healthcare identified factors unique to dentistry [29]. Taking account of context is recognised key to the successful translation of interventions to new settings, and for dentistry these unique factors [30] related to the surgical nature of the profession, where infections are usually readily amenable to procedures without the need for antibiotics [7], and where surgical prophylaxis is often the most common reason for dentists to prescribe antibiotics [31].

Despite antimicrobial stewardship aiming to optimise the use of these medications, antimicrobial stewardship programmes (ASPs) across human healthcare have tended to focus simply on reduced prescribing and ignoring any increase in adverse outcomes or patient feedback [32, 33]. This was also the approach taken by Elouafkaoui et al. [23] in relation to the dental audit and feedback intervention. A recent systematic review of the impact of ASPs on antibiotic use found a 10% reduction in antibiotic prescribing and a 28% reduction in the consumption of antibiotics, as well as an improvement in the quality of prescribing in relation to antibiotics in the WHO Watch group [34].

Prescribing decisions are complex. Studies have identified more than 30 potentially modifiable factors associated with the decision whether to prescribe antibiotics in therapeutic and prophylactic dental scenarios, including system issues such as healthcare context (e.g., expectation/pressure towards reduced prescribing) and incentives (e.g., remuneration [29, 31, 35, 36, 37]). Knowledge and guidelines are also identified as important, but as highlighted by Sneddon et al. [31], they are necessary but not sufficient to achieve behaviour change [38]. Unsurprisingly, our study shows that an intervention tackling system level issues (via audit & feedback) [23] had more impact of dental antibiotic prescribing than ones focused on improving knowledge about guidelines through education [22, 24].

Audit & feedback as employed by Elouafkaoui et al. [23] relies on national surveillance systems to routinely collecting reliable data about antibiotic use. The WHO global action plans on AMR highlighted the importance of such surveillance at a national level [39]. Many national action plans reflect this priority, although few have highlighted the challenge of such a system for dentistry [28, 40]. To ensure optimisation of antibiotic use, rather than just reduction, these systems also need to include information about clinical indications. In 2016, England's National Institute for Health & Care Excellence (NICE) quality standard on antimicrobial stewardship included a developmental statement to align hospital‐based and dental prescribers with those in general practice: ‘Prescribers in secondary and dental care use electronic prescribing systems that link indication with antimicrobial prescription’ [41]. Significant challenges exist to delivery of such surveillance systems across dentistry and the 2024 revision of the UK national action plan on AMR again identified the challenge of developing such a system for dentistry [40]. The authors are unaware of any nation which routinely collects data about the antibiotic regimen and indication associated with dental prescriptions. Whilst waiting for policy makers to deliver such national surveillance systems, more local approaches for collecting data in dental practices and educating prescribers via face‐to‐face approaches as demonstrated by Seager et al. [22] and Chehabeddine et al. [24] should be introduced. The next challenge is to identify the workforce best placed to deliver this. Across healthcare, antimicrobial stewardship is positioned alongside infection prevention and control (IPC), based on a model of communicable diseases. Within dentistry, IPC is general led by members of the oral health team other than dentists, whose role is to prevent the spread of infection within the clinic, for example through decontamination and disinfection of instruments. However, prescribing is only within the scope of practice for dentists, so other members of the team have much less education and training in this area. Relying on dentists to lead on antibiotic audits and deliver courses about antimicrobial prescribing, resistance and stewardship is, therefore, ideal albeit more expensive than being led by other healthcare professionals. To overcome this, a self‐audit approach has been introduced in England [42]. The complex algorithm to identify appropriate (guideline congruent) antibiotic prescribing has, however, been confusing for those not fully conversant with the guidelines. Clinical decision support tools integrated into practice management software have shown promise to assist dentists optimise their antibiotic prescribing [12]. Heterogeneity in the outcomes employed to measure the effect of the dental AMS interventions meant that the results could not be easily synthesised. This correlates with the findings of a systematic review of outcome measures for ABS interventions across primary care (including but not limited to dentistry), which highlighted the need to measure standardised patient outcomes and antibiotic use [43]. To enable a meaningful comparison of the results of future studies, we recommend the internationally standardised core outcome set for dental ABS should be reported in future studies [15]. However, the evaluation of dental ABS interventions should go beyond simply measuring its effectiveness at achieving its intended outcome [44]. Full evaluation should be designed to provide insight into a wider range of issues such as how the intervention works (through process evaluation), its cost‐effectiveness, interactions with the context in which it is being implemented, and its contribution to changing systems. By providing this sort of evidence, dental ABS evaluation studies can provide support to real world decision making [44].

A strength of this study is its strict criteria for inclusion and risk of bias assessment. However, this has highlighted the weakness of the evidence base for dental ABS and resulted in the exclusion of at least 15 further studies of dental AMS interventions due to study design issues, such as employing a before and after (also known as pre‐post) design without a comparator group [12, 14, 27, 45, 46, 47, 48, 49, 50, 51, 52, 53, 54, 55, 56]. With just three studies included which were too heterogenous for meta‐analysis, a weakness of the study was the need for narrative synthesis which provides no information about the size of the overall effect.

## 5 Conclusion

Although a wide variety of dental antibiotic stewardship interventions have been reported in the literature, only three have been evaluated using a randomised design and the quality of evidence provided is variable (from high to very low certainty). The significant contribution of dentistry towards inappropriate antibiotic prescribing globally underscores the need for effective interventions to improve the use of antibiotics, ensuring that they are used only when strictly necessary. To improve confidence in the body of evidence underpinning dental antibiotic stewardship interventions, well‐powered, robust randomised controlled trials with adequate follow‐up and reporting the core outcome set are required. The researchers of dental ABS should be encouraged and supported to fully evaluate their interventions, in line with the latest guidance, including process evaluation.

## Author Contributions

L.T. contributed to the conception of the work, the analysis, interpretation of the data and drafted the manuscript; C.L. contributed to the acquisition and analysis, and drafted the manuscript; M.M. contributed to the acquisition and analysis, and drafted the manuscript; A.A. contributed to the analysis and drafted the manuscript; H.K. contributed to the acquisition and analysis, and drafted the manuscript; K.B. contributed to the conception of the work and drafted the manuscript; W.T. contributed to the conception and design of the work, the acquisition, analysis and interpretation of the data, and drafted the manuscript. All authors read and approved the final manuscript.

## Ethics Statement

The authors have nothing to report.

## Consent

The authors have nothing to report.

## Conflicts of Interest

The authors declare no conflicts of interest.

## Supporting information


Data S1.

## Data Availability

All data generated or analysed during this study are included in the published article and its (Data S1).
