# Supplementary material for: A Systematic Review of Dental Antibiotic Stewardship Interventions
Source: Community Dent Oral Epidemiol. 2024 Oct 14;53(3):245–55. doi: 10.1111/cdoe.13009 (PMC12064870; doi:10.1111/cdoe.13009)
Supplement: Supplementary file 1 — Data S1. [file CDOE-53-245-s001.docx]

**Supplementary Data 1 - Search Strategy**

MEDLINE: [((Antibiotic* OR anti-bacterial* OR antimicrob*) ADJ3 (prescri* OR stewardship)).mp OR exp antibiotic prophylaxis/ OR exp antimicrobial stewardship/] AND [(dentist* OR dental care OR dentistry OR dental surgeon* OR dental practi*).mp OR exp Dentistry/] AND [(intervention OR pilot OR audit OR educat* OR train*).mp OR exp clinical trial/ OR exp observational study/] - Limit to humans

EMBASE: [(Antibiotic* OR anti-bacterial* OR antimicrobial) ADJ3 (prescri* OR stewardship).mp OR exp antibiotic prophylaxis/ OR exp antimicrobial stewardship/} AND [(dentist* OR dental care OR dental surge* OR dental practi*).mp OR exp dentistry/] AND [(intervention OR pilot OR audit OR educat* OR train*).mp OR exp clinical trial/ OR exp observational study/] - Limit to humans

Dentistry and Oral Sciences Source: [Antibiotic prescri* OR anti-bacterial prescri* OR antimicrobial prescri* OR antibiotic stewardship OR antimicrobial stewardship] AND [dentist* OR dental care OR dentistry OR dental surgeon* OR dental practi*] AND [intervention* OR stud* OR trial* OR pilot OR audit OR educat* OR train*]

Cochrane Oral Health Group Trials Register: [Antibiotic prescri* OR anti-bacterial prescri* OR antimicrobial prescri* OR antibiotic stewardship OR antimicrobial stewardship] AND [dentist* OR dental care OR dentistry OR dental surgeon* OR dental practi*] AND [intervention* OR stud* OR trial* OR pilot OR project OR research OR stewardship* OR audit OR educat* OR train*]

Cochrane Central Register of Controlled Trials: [Antibiotic prescri* OR anti-bacterial prescri* OR antimicrobial prescri* OR antibiotic stewardship OR antimicrobial stewardship] AND [dentist* OR dental care OR dentistry OR dental surgeon* OR dental practi*] AND [intervention* OR stud* OR trial* OR pilot OR project OR research OR stewardship* OR audit OR educat* OR train*]

ISRCTN: [Antibiotic OR antimicrobial] AND [Dentist OR Dental]

ICTRP: [Antibiotic OR antimicrobial] AND [Dentist OR Dental]

NIH Trials Register: [Antibiotic OR antimicrobial] AND [Dentist OR Dental]

**Supplementary Table 2:** **Summary of excluded studies**

| **Study** | **Title** | **Study design/Report type** | **Reason for exclusion** |
| --- | --- | --- | --- |
| Steed and Gibson 1997 | An audit of antibiotic prescribing in general dental practice | Before-and-after study | Wrong study design |
| Thomas and Hill, 1997 | An audit of antibiotic prescribing in third molar surgery. | Before-and-after study | Wrong study design |
| Palmer et al, 1998 | An investigation of antibiotic prescribing by general dental practitioners: a pilot study. | Point prevalence study | Wrong study design |
| Palmer et al, 2001 | Can audit improve antibiotic prescribing in general dental practice? | Before-and-after study | Wrong study design |
| Palmer et al, 2001 | Paediatric antibiotic prescribing by general dentists in England | Point prevalence study | Wrong study design |
| Pendlebury, 2001 | Clinical audit: a study to improve antibiotic prescribing. | Commentary about Palmer et al, 2001 | Wrong report type |
| Chate et al, 2006 | The impact of clinical audit on antibiotic prescribing in general dental practice. | Before-and-after study | Wrong study design |
| Martin et al, 2006 | Antibiotic prescribing for acute dental pain. | Commentary about Seager et al, 2006 | Wrong report type |
| Rauniar et al, 2012 | Effectiveness of an educational feedback intervention on drug prescribing in dental practice. | Before-and-after study | Wrong study design |
| Chopra et al, 2014 | An audit of antimicrobial prescribing in an acute dental care department | Before-and-after study | Wrong study design |
| Walshe et al, 2014 | Clinical audit on antibiotic prescribing in the oral surgery department following general anaesthetic and intravenous sedation outpatient procedures at Guy’s dental hospital. | Before-and-after study | Wrong study design |
| Zahabiyoun et al, 2015 | Improving Knowledge of General Dental Practitioners on Antibiotic Prescribing by Raising Awareness of the Faculty of General Dental Practice (UK) Guidelines | Before-and-after study | Wrong study design |
| Cope et al, 2016 | Antimicrobial prescribing by dentists in Wales, UK; findings of the first cycle of a clinical audit | Point prevalence study | Wrong study design |
| Gross et al, 2019 | Successful implementation of an antibiotic stewardship program in an academic dental practice | Before-and-after study | Wrong study design |
| Caniff et al, 2020 | Post-extraction infection and antibiotic prescribing among veterans receiving dental extractions. | Retrospective cohort | Wrong outcome |
| Jacobsen et al, 2020 | Prescription of antibiotics – a quality project within the public dental services in Västra Götaland region. | Before-and-after study | Wrong study design |
| Karaben et al, 2020 | Observation and educational intervention to modify the prescription of antibiotics in an Institute Social in the city of Corrientes, Argentina. | Before-and-after study | Wrong study design |
| Scott et al, 2020 | Evaluating the effectiveness of behaviour change theory-based training for reducing antibiotic prescribing by NHS dentists working in NHS primary care dental practices | Protocol | Wrong report type |
| Teoh et al, 2021 | Improvement of dental prescribing practices using education and a prescribing tool: A pilot intervention study | Before-and-after study | Wrong study design |
| George et al, 2022 | Influence of CRP on Antibiotics Prescription Pattern for Dental Infections: A Prospective Interventional Study | Before-and-after study | Wrong study design |
| Goff et al, 2022 | Private Practice Dentists Improve Antibiotic Use After Dental Antibiotic Stewardship Education From Infectious Diseases Experts | Before-and-after study | Wrong study design |
| Wan-Lin et al, 2022 | A clinical audit and impact of interventions on antibiotic prescribing practices at a public dental primary care clinic. | Before-and-after study | Wrong study design |

**Supplementary Data 3**

**COCHRANE RISK OF BIAS 2 TOOL**

**Key to acronyms:**

**Y: yes**

**PY: probably yes**

**N: no**

**NI: not included**

**NA: not applicable**

**Assessor: LT**

**Basic information**

| **Date** | **Unique ID** | **Study ID** | **Reference** | **Experimental** | **Comparator** | **Outcome** | **Results** | **Aim** | **Effect of adhering to intervention** | **Weight** | **Sources** |
| --- | --- | --- | --- | --- | --- | --- | --- | --- | --- | --- | --- |
| 14/07/2023 | Study 1 | Chehabeddine 2022 | Chehabeddine 2022 | Educational intervention | control group | mean percentage of antibiotics prescribed; prescriptions compliant with indication | Table 3 | assignment to intervention (the 'intention-to-treat' effect) | NA | 1 | Journal article(s) |
| 14/07/2023 | Study 2 | Seager 2006 | Seager 2006 | Educational outreach; guideline | control group | 1) all antibiotic prescriptions and 2) inappropriate antibiotic prescriptions. Inappropriate antibiotic prescribing was defined as the provision of an antibiotic to a patient who did not present with a symptom indicative of spreading infection. | Table 3 | assignment to intervention (the 'intention-to-treat' effect) | NA |  | Journal article(s) |
| 14/07/2023 | Study 3 | Elouafkaoui 2016 | Elouafkaoui 2016 | Individualised A & F | control group | Total number of antibiotic items dispensed per 100 NHS treatment claims over the 12 month post-delivery of the baseline audit and feedback. | Overall adjusted effect size of -0.47 (-0.85, -0.09) fewer | assignment to intervention (the 'intention-to-treat' effect) | NA |  | Journal article(s); Trial protocol |

**Domain 1a. Randomisation process**

| **Study ID** | **Study** | **1a.1** | **1a.2** | **Note for 1a.1&1a.2** | **1a.3** | **Note for 1a.3** | **1a.0 algorithm result** | **1a.0 Assessor's Judgement** |
| --- | --- | --- | --- | --- | --- | --- | --- | --- |
| Chehabeddine 2022 | Study 1 | PY | NI | Randomly allocated computer generated list using Excel 2016 | PN |  | Some concerns | Some concerns |
| Seager 2006 | Study 2 | Y | PY | stratified randomisation using a computer program developed in house stratification done remotely by Uni department | N |  | Low | Low |
| Elouafkaoui 2016 | Study 3 | Y | PY | computer generated, stratified randomisation. Even allocation. Presumed concealed allocation by remote third party | N | Prescribing rates similar across groups at baseline | Low | Low |

**Domain 1b: Risk of bias arising from the timing of identification or recruitment of participants**

| **Study ID** | **1b.1** | **Note for 1b.1** | **1b.2** | **1b.3** | **Note for 1b.3** | **1b.0 Algorithm result** | **1b.0 Assessor's Judgement** |
| --- | --- | --- | --- | --- | --- | --- | --- |
| Chehabeddine 2022 | N | "data were collected from all their patients who sought consultation over 2 months" but some clusters in which no participants were recruited. | PN | N |  | Low | Low |
| Seager 2006 | N | individual participants were not recruited at all but were identified before randomization' every adult presenting with acute dental pain during the trial period.' but some clusters where participants were not recruited. | PN | PN | Table 1 | Low | Low |
| Elouafkaoui 2016 | PY | Individuals not recruited, but identified before randomisation - all treatment claims data during the trial period. | NA | N |  | Low | Low |

**Domain 2. Deviations from intended interventions**

| **Study ID** | **2.1a** | **Note for 2.1a** | **2.1b (2.1)** | **2.2** | **Note for 2.1b&2.2** | **2.3** | **2.4** | **2.5** | **2.6** | **2.7** | **2.0 Algorithm result** | **2.0 Assessor's Judgement** |
| --- | --- | --- | --- | --- | --- | --- | --- | --- | --- | --- | --- | --- |
| Chehabeddine 2022 | PN | No indication that participants were informed. | NA | Y | Due to nature of the intervention | PN | NA | NA | PY | NA | Low | Low |
| Seager 2006 | PN | No indication that participants were informed. | NA | Y | Due to nature of the intervention. Intervention at dentist level. | PN | NA | NA | PY | NA | Low | Low |
| Elouafkaoui 2016 | PN | No indication that participants were informed. | NA | Y | Due to nature of the intervention | PN | NA | NA | PY | NA | Low | Low |

**Domain 3. Missing outcome data**

| **Study ID** | **3.1a** | **3.1b** | **3.2** | **3.3** | **3.4** | **Note for 3.3&3.4** | **3.0 Algorithm result** | **3.0 Assessor's judgement** |
| --- | --- | --- | --- | --- | --- | --- | --- | --- |
| Chehabeddine 2022 | PY | NI | N | PY | PN |  | Some concerns | Some concerns |
| Seager 2006 | Y | NI | N | PY |  | Possible that loss to follow-up was related to the outcome (antibiotics prescribed) Differences in the number of questionnaires returned by cluster, and the number of clusters returning no questionnaires. | High | High |
| Elouafkaoui 2016 | Y | PY | NA | NA | NA |  | Low | Low |

**Domain 4. Measurement of the outcome**

| **Study ID** | **4.1** | **4.2** | **4.3a** | **Note for 4.3a** | **4.3b** | **Note for 4.3b** | **4.4** | **Note for 4.4&4.5** | **4.0 Algorithm result** | **4.0 Assessor's Judgement** |
| --- | --- | --- | --- | --- | --- | --- | --- | --- | --- | --- |
| Chehabeddine 2022 | N | N | PY | PI visited clinics weekly to collect proformas for outcome assessment | PY |  | PN | No information concerning blinding but number of antibiotic prescriptions and appropriateness unlikely to be influenced by knowledge of condition | Low | Low |
| Seager 2006 | N | PN | PY |  | NI | Unclear - 'information on antibiotic prescribing was not specifically requested and practitioners were not told that the study was about antibiotic prescribing | PN |  | Low | Low |
| Elouafkaoui 2016 | N | N | Y |  | N | Outcome assessor was trial statistician who was blinded to allocation | NA |  | Low | Low |

**Domain 5. Selection of the reported result**

| **Study ID** | **5.1** | **Note for 5.1** | **5.2** | **5.3** | **5.0 Algorithm result** | **5.0 Assessor's Judgement** |
| --- | --- | --- | --- | --- | --- | --- |
| Chehabeddine 2022 | NI | No trial registry information | N | N | Some concerns | Some concerns |
| Seager 2006 | NI |  | N | N | High | High |
| Elouafkaoui 2016 | NI |  | N | N | Low | Low |

**Domain 6. Overall Bias**

| **Study ID** | **Algorithm's overall Judgement** | **Assessor's overall Judgement** |
| --- | --- | --- |
| Chehabeddine 2022 | High | High |
| Seager 2006 | High | High |
| Elouafkaoui 2016 | Low | Low |

**Assessor: MM**

**Basic information**

| **Date** | **Unique ID** | **Study ID** | **Reference** | **Experimental** | **Comparator** | **Outcome** | **Results** | **Aim** | **Effect of adhering to intervention** | **Weight** | **Sources** |
| --- | --- | --- | --- | --- | --- | --- | --- | --- | --- | --- | --- |
| 14/07/2023 | Study 1 | Chehabeddine 2022 | Chehabeddine 2022 | Educational intervention | control group | mean percentage of antibiotics prescribed; prescriptions concordance with guidelines by indication (clinical scenario) | Table 3 | assignment to intervention (the 'intention-to-treat' effect) | NA | 1 | Journal article(s) |
| 14/07/2023 | Study 2 | Seager 2006 | Seager 2006 | Educational outreach; guideline | control group | 1) all antibiotic prescriptions and 2) inappropriate antibiotic prescriptions, in accordance with the correct indication (not for spreading infections). | Table 3 | assignment to intervention (the 'intention-to-treat' effect) | NA |  | Journal article(s) |
| 14/07/2023 | Study 3 | Elouafkaoui 2016 | Elouafkaoui 2016 | Individualised A & F | control group | Total number of antibiotic items dispensed per 100 NHS treatment claims over the 12 month post-delivery of the baseline A and F | Overall adjusted effect size of -0.47 (-0.85, -0.09) fewer | assignment to intervention (the 'intention-to-treat' effect) | NA |  | Journal article(s); Trial protocol |

**Domain 1a. Randomisation process**

| **Study ID** | **Study** | **1a.1** | **1a.2** | **Note for 1a.1&1a.2** | **1a.3** | **Note for 1a.3** | **1a.0 algorithm result** | **1a.0 Assessor's Judgement** |
| --- | --- | --- | --- | --- | --- | --- | --- | --- |
| Chehabeddine 2022 | Study 1 | PY | NI | Randomly allocated computer generated list; used Excel | PN |  | Some concerns | Some concerns |
| Seager 2006 | Study 2 | Y | PY | stratified randomisation using a computer program; Uni department conducted the stratification | N |  | Low | Low |
| Elouafkaoui 2016 | Study 3 | Y | PY | computer generated randomisation | N | Prescribing rates similar across groups at baseline | Low | Low |

**Domain 1b: Risk of bias arising from the timing of identification or recruitment of participants**

| **Study ID** | **1b.1** | **Note for 1b.1** | **1b.2** | **1b.3** | **Note for 1b.3** | **1b.0 Algorithm result** | **1b.0 Assessor's Judgement** |
| --- | --- | --- | --- | --- | --- | --- | --- |
| Chehabeddine 2022 | N | "data were collected from all their patients who sought consultation over 2 months" | PN | N |  | Low | Low |
| Seager 2006 | N | individual participants were not recruited at all but were identified before randomization'. Some groups did not recruit participants | PN | PN | Table 1 | Low | Low |
| Elouafkaoui 2016 | PY | Individuals not recruited, but identified before randomisation - all treatment claims data during the trial period. | NA | N |  | Low | Low |

**Domain 2. Deviations from intended interventions**

| **Study ID** | **2.1a** | **Note for 2.1a** | **2.1b (2.1)** | **2.2** | **Note for 2.1b&2.2** | **2.3** | **2.4** | **2.5** | **2.6** | **2.7** | **2.0 Algorithm result** | **2.0 Assessor's Judgement** |
| --- | --- | --- | --- | --- | --- | --- | --- | --- | --- | --- | --- | --- |
| Chehabeddine 2022 | PN | No indication that participants were informed. | NA | Y | Due to nature of the intervention | PN | NA | NA | PY | NA | Low | Low |
| Seager 2006 | PN | No indication that participants were informed. | NA | Y | Due to nature of the intervention. Intervention at dentist level. | PN | NA | NA | PY | NA | Low | Low |
| Elouafkaoui 2016 | PN | No indication that participants were informed. | NA | Y | Due to nature of the intervention | PN | NA | NA | PY | NA | Low | Low |

**Domain 3. Missing outcome data**

| **Study ID** | **3.1a** | **3.1b** | **3.2** | **3.3** | **3.4** | **Note for 3.3&3.4** | **3.0 Algorithm result** | **3.0 Assessor's judgement** |
| --- | --- | --- | --- | --- | --- | --- | --- | --- |
| Chehabeddine 2022 | PY | NI | N | PY | PN |  | Some concerns | Some concerns |
| Seager 2006 | Y | NI | N | PY |  | Loss to follow up (may relate to quantity of antibiotics prescribed) Differences in the number of questionnaires returned by each group | High | High |
| Elouafkaoui 2016 | Y | PY | NA | NA | NA |  | Low | Low |

**Domain 4. Measurement of the outcome**

| **Study ID** | **4.1** | **4.2** | **4.3a** | **4.3b** | **Note for 4.3b** | **4.4** | **4.0 Algorithm result** | **4.0 Assessor's Judgement** |
| --- | --- | --- | --- | --- | --- | --- | --- | --- |
| Chehabeddine 2022 | N | N | PY | PY |  | PN | Low | Low |
| Seager 2006 | N | PN | PY | NI |  | PN | Low | Low |
| Elouafkaoui 2016 | N | N | Y | N | Trial statistician was blinded | NA | Low | Low |

**Domain 5. Selection of the reported result**

| **Study ID** | **5.1** | **Note for 5.1** | **5.2** | **5.3** | **5.0 Algorithm result** | **5.0 Assessor's Judgement** |
| --- | --- | --- | --- | --- | --- | --- |
| Chehabeddine 2022 | NI | No trial registry information | N | N | Some concerns | Some concerns |
| Seager 2006 | NI |  | N | N | High | High |
| Elouafkaoui 2016 | NI |  | N | N | Low | Low |

**Domain 6. Overall Bias**

| **Study ID** | **Algorithm's overall Judgement** | **Assessor's overall Judgement** |
| --- | --- | --- |
| Chehabeddine 2022 | High | High |
| Seager 2006 | High | High |
| Elouafkaoui 2016 | Low | Low |

**Supplementary Table 4: GRADE Assessment**

| **Summary of findings:** | | | | | | | |
| --- | --- | --- | --- | --- | --- | --- | --- |
| **Dental Antibiotic Stewardship compared to Standard Care for Antibiotic Prescriptions** | | | | | | | |
| **Patient or population: Antibiotic Prescriptions**  **Setting: in dental clinics**  **Intervention: Dental Antibiotic Stewardship**  **Comparison: Standard Care** | | | | | | | |
| Outcomes | | **Anticipated absolute effects^*^** (95% CI) | | Relative effect (95% CI) | № of participants (studies) | Certainty of the evidence (GRADE) | Comments |
|  |  | **Risk with Standard Care** | **Risk with Dental Antibiotic Stewardship** |  |  |  |  |
| Inappropriate Antibiotic Prescribing for acute dental pain with no intervention, or antibiotic stewardship with educational leaflets (Inappropriate Antibiotics Precribing with Education leaflets) assessed with: inappropriate prescription numbers (Seager et al) | | 180 per 1,000 | **174 per 1,000** (159 to 186) | **RR 0.968** (0.883 to 1.035) | 941 (1 RCT) | ⨁◯◯◯ Very low^a,b^ | The evidence is very uncertain about the effect of dental Antibiotic Stewardship on inappropriate Antibiotic Prescribing for acute dental pain with no intervention, or antibiotic stewardship with educational leaflets. |
| Inappropriate Antibiotic Prescribing for acute dental pain with no intervention, or antibiotic stewardship with pharmacist academic briefing and educational leaflets (Inappropriate Antibiotic Prescribing with Pharamacist and leaflets (Seager et al)) assessed with: % of prescription | | 180 per 1,000 | **154 per 1,000** (142 to 170) | **RR 0.860** (0.792 to 0.944) | 1046 (1 RCT) | ⨁⨁◯◯ Low^a^ | The evidence suggests that dental Antibiotic Stewardship results in a large reduction in inappropriate Antibiotic Prescribing for acute dental pain with no intervention, or antibiotic stewardship with pharmacist academic briefing and educational leaflets. |
| Antibiotics Dispensed per 100 claims by dentists with and without audit and feedback (A &F) (Rate of Antibiotic Prescribing (Elouafkaoui et al)) assessed with: Antibiotics Dispensed per 100 claims | | The rate of antibiotic prescribing by dentists receiving an A&F intervention was reduced from 8.5 items per 100 NHS treatment claims at baseline to 7.5 items per 100 NHS treatment claims at follow-up. Dentists in the control group also reduced antibiotic prescribing from 8.3 items per 100 NHS treatment claims to 7.9 items per 100 NHS treatment claims, giving an overall adjusted effect size of 0.47 (95% CI 0.09 to 0.85) fewer antibiotic items per 100 NHS treatment claims. | |  | 2566 (1 RCT) | ⨁⨁⨁⨁ High^a^ | Dental Antibiotic Stewardship likely results in a large increase/reduction in antibiotics Dispensed per 100 claims by dentists with and without audit and feedback (A &F). |
| ***The risk in the intervention group** (and its 95% confidence interval) is based on the assumed risk in the comparison group and the **relative effect** of the intervention (and its 95% CI).  **CI:** confidence interval; **RR:** risk ratio | | | | | | | |
| **GRADE Working Group grades of evidence** **High certainty:** we are very confident that the true effect lies close to that of the estimate of the effect. **Moderate certainty:** we are moderately confident in the effect estimate: the true effect is likely to be close to the estimate of the effect, but there is a possibility that it is substantially different. **Low certainty:** our confidence in the effect estimate is limited: the true effect may be substantially different from the estimate of the effect. **Very low certainty:** we have very little confidence in the effect estimate: the true effect is likely to be substantially different from the estimate of effect. | | | | | | | |

#### Explanations

a. according to RoB 2 assessment

b. Risk Ratio confidence intervals show high variation
